# Intercellular communication analysis of the human retinal pigment epithelial and choroidal cells predicts pathways associated with aging, cellular senescence and age-related macular degeneration

**DOI:** 10.3389/fnagi.2022.1016293

**Published:** 2022-11-03

**Authors:** Dhanach Dhirachaikulpanich, Cyril Lagger, Kasit Chatsirisupachai, João Pedro de Magalhães, Luminita Paraoan

**Affiliations:** ^1^Ocular Molecular Biology and Mechanisms of Disease Group, Institute of Life Course and Medical Sciences, University of Liverpool, Liverpool, United Kingdom; ^2^Faculty of Medicine, Siriraj Hospital, Mahidol University, Bangkok, Thailand; ^3^Integrative Genomics of Ageing Group, Institute of Life Course and Medical Sciences, University of Liverpool, Liverpool, United Kingdom

**Keywords:** RPE, choroid, aging, senescence, VEGF, BMP, tenascin, AMD

## Abstract

The retinal pigment epithelium (RPE) and the choroid are ocular tissues with fundamental roles in supporting neuroretinal function. The pathogenesis of age-related macular degeneration (AMD), a leading cause of irreversible blindness for which aging is the highest risk factor is closely linked with progressive impairment of various functions of these tissues. Cellular senescence, marked by cell cycle arrest and secretion of proinflammatory factors, is known to be associated with aging and has been proposed as a potential driver of AMD. Here, we investigated the role played by intercellular communication in the RPE/choroid within the context of aging, senescence and AMD. We inferred cell–cell interactions in the RPE/choroid by applying CellChat and scDiffCom on a publicly available scRNA-seq dataset from three human donors with and without AMD. We identified age-regulated ligand and receptor genes by using limma on a separate publicly available bulk microarray dataset providing RPE/choroid samples at multiple time points. Cellular senescence was investigated by assigning a score to each cell and each sample of these scRNA-seq and microarray datasets, respectively, based on the expression of key signature genes determined by a previous senescence meta-analysis. We identified VEGF-, BMP-and tenascin-mediated pathways supporting some of the strongest cell–cell interactions between RPE cells, fibroblasts and choroidal endothelial cells and as strong intercellular communication pathways related to both aging and senescence. Their signaling strength was enhanced between subpopulations of cells having high senescence scores. Predominant ligands of these pathways were upregulated with age whereas predominant receptors were downregulated. Globally, we also observed that cells from AMD samples presented slightly bigger senescence scores than normal cells and that the senescence score positively correlated with age in bulk samples (*R* = 0.26, value of *p* < 0.01). Hence, our analysis provides novel information on RPE/choroid intercellular communication that gives insights into the connection between aging, senescence and AMD.

## Introduction

Age-related macular degeneration (AMD) is the leading cause of irreversible blindness in adults in developed countries (Wong et al., 2014), which has been linked to numerous genetical and environmental risk factors and most importantly age (Group et al., 2012; Wong et al., 2014). However, the molecular mechanisms by which aging contributes to AMD pathogenesis are far from being fully characterized. One hypothesis suggests cellular senescence of the retinal pigment epithelium (RPE), the cell monolayer with essential role in supporting the function of the neuroretina, as a key process promoting the development of AMD (Kozlowski, 2012). Cellular senescence impairs the ability of self-renewal – a biological process that is particularly important for the mitotically inactive RPE – and contributes to an increased inflammatory phenotype, mostly through the secretion of inflammatory cytokines and proteases, collectively defined as the senescence-associated secretory phenotype (SASP) (Kozlowski, 2012; Wang et al., 2019; Basisty et al., 2020). Age-related factors like increased oxidative stress, altered proteostasis and DNA damage are thought to promote senescence in several age-related neurodegenerative diseases such as Alzheimer’s disease and Parkinson’s disease (Kritsilis et al., 2018; Martinez-Cue and Rueda, 2020). An increase in senescent cells in aged RPE and choroid tissues has also been reported (Chaum et al., 2015; Cabrera et al., 2016) consistent with altered cell signaling known to promote chronic inflammation and RPE cellular dysfunction (Cao et al., 2013). Senescent RPE cells likely influence neighboring cells, potentially contributing to the development of AMD characteristics such as increased choroidal endothelial stiffness and membrane attack complex deposition (Cabrera et al., 2016; Lee et al., 2021). Although an ongoing effort is trying to connect aging, cellular senescence and the pathogenesis of AMD, the molecular mechanisms underlying their relationship are still not well established (Blasiak et al., 2017; Sreekumar et al., 2020).

The RPE and the choroid form a system that plays crucial roles in the normal function of the neuroretina as well as in the pathology of AMD (Strauss, 2005). It supports the neuroretinal metabolism by supplying nutrients and facilitating the removal of waste. The junctional complex of the RPE is critical for the blood–brain barrier while the tissue itself acts as a secretory machinery to support the communication between the choroid and the retina (Paraoan et al., 2020). During AMD, lipoproteinaceous and other extracellular debris accumulate between the RPE and the choroid, leading to the formation of drusen (Ardeljan and Chan, 2013). Previous research investigating how this system ages focused on the RPE or the choroid alone (Paraoan et al., 2000; Kay et al., 2013, 2014; Porter et al., 2019; Sharif et al., 2019; Voigt et al., 2019, 2020; Dhirachaikulpanich et al., 2020; Butler et al., 2021; Saptarshi et al., 2021). However, given that the RPE/choroid system is a complex microenvironment composed of many cell types functioning together, including RPE cells, endothelial cells, fibroblasts and immune cells (Voigt et al., 2021, 2022), the communication between the cellular components of the RPE/choroid needs also to be characterized.

The advent of single-cell RNA sequencing (scRNA-seq) and the development of cell–cell communication software now enables the investigation of the interactions between different cell types in a tissue (Armingol et al., 2021). In this context, intercellular communication (ICC) is defined as a set of signaling interactions involving secreted proteins (ligands) from one cell type and membrane-bound proteins (receptors) from another (or the same) cell type (Jin et al., 2021). Inferring such intercellular communication patterns, including secreted protein crosstalk and extracellular matrix receptor interactions, could prove useful to identify key signaling pathways in normal and disease conditions (Armingol et al., 2021; Jin et al., 2021). This type of analysis has been applied to a variety of diseases, including COVID-19 (He et al., 2021; Yang et al., 2021), wound healing (Hu et al., 2021), inflammatory bowel disease (Corridoni et al., 2020). However, no study has yet investigated intercellular communication of the RPE/choroid in the context of aging, senescence and AMD. For this purpose here we leveraged publicly available microarray and scRNA-seq datasets of the RPE/choroid to investigate cell–cell signaling altered by aging and senescence in normal and AMD tissues. Our analysis predicts three specific age-related pathways (VEGF, BMP and tenascin) that are enriched in interactions taking place between a set of fibroblasts, RPE cells and endothelial cells characterized by high expression of senescence signature genes. Although the scarcity of the currently available data does not allow to firmly establish how these pathways differ between AMD and normal patients, we independently observe that AMD samples have a higher expression of senescence signature genes compared to normal samples. Together, our results support the notion that cellular senescence plays an important role in the aging of the RPE/choroid microenvironment and AMD pathogenesis.

## Materials and methods

### Single-cell RNA-seq data acquisition and processing

We retrieved publicly available scRNA-seq RPE/choroid data (GSE135922) containing samples from three human donors, including two normal eyes and one uncharacterized neovascular AMD eye (Voigt et al., 2019). This dataset was downloaded as a Seurat object through the human cell atlas galaxy portal (Moreno et al., 2021; Papatheodorou et al., 2020). The subsequent analysis was done in R (version 4.0.2). Cells were filtered out if they had unique gene counts lower than 300 or more than 7,000. Log normalization was performed with a scale factor of 10,000 using Seurat (version 4.0.1). The Seurat functions FindNeighbors and FindClusters were used to cluster the cells based on the first 10 principal components returned by the function RunPCA. For visualization purposes, UMAP dimensional reduction was performed with the function RunUMAP using the same 10 principal components (Hao et al., 2021). The gene markers’ identifier of each cell cluster was done with the function FindAllMarkers. These markers were used to annotate each cluster by cell types similarly to Voigt et al. (2019).

### Intercellular communication analysis

Intercellular communication (ICC) analysis was performed on the pre-processed Seurat object described above with two R packages relying on different detection methods: CellChat (Jin et al., 2021) and scDiffCom (Lagger et al., 2021). Both algorithms combine the cell type-specific expression of known ligand and receptor genes with permutation tests to assess the biological relevance of potential cell–cell interactions (CCIs). They differ in the way they quantify the strength of these interactions (respectively referred to as “communication probability” and “CCI score”) and in their internal statistical implementations. scDiffCom was also specifically designed to perform differential ICC analysis between biological conditions, but this functionality was not used in the current study because of the low sample size of the aforementioned scRNA-seq dataset. Default settings of CellChat (version 1.0.0) were used as recommended (Jin et al., 2021). scDiffCom (version 0.2.3) was used in “detection-mode-only” with default parameters, except for the number of permutations that was set to 10,000 (instead of 1,000) and the “quantile expression threshold” that was set to 0 (instead of 0.2). The latter parameter is used by scDiffCom to filter out statistically significant but lowly expressed CCIs. As such filtering is not performed by CellChat by default, we disabled it in this analysis to obtain more similar results. Detection of CCIs by both packages crucially depends on the prior database of curated ligand-receptor interactions (LRI) they rely on. Such LRIs can be pairs of genes (e.g., AMD: CALCR) or include additional factors to describe heteromeric complexes (e.g., ANGPT2:ITGA1-ITGB1). CellChat manually created a curated list of 1,939 human LRIs principally based on knowledge extracted from the KEGG database (Kanehisa et al., 2016). However, scDiffCom relies on a larger collection of 4,785 LRIs obtained by combining seven LRI databases that had been manually curated by previous studies, including CellChat itself. As scDiffCom has therefore the potential to detect more CCIs than CellChat, we took this factor into account when comparing their results.

Network analysis to compute centrality scores of outgoing, incoming or mediator communication pathways was performed with CellChat built-in function netAnalysis_computeCentrality. The determination of the dominant and most relevant pathways was performed by exploring and comparing network results manually and by using either visualization tools provided by CellChat (e.g., netAnalysis_signalingRole_network) or custom scripts for scDiffCom results.

### Differential analysis of ligand/receptor genes in aging RPE/choroid microarray datasets

Transcriptomic microarray data were retrieved from GSE29801 (Newman et al., 2012). This dataset contained 96 post-mortem RPE/choroid samples with no previous ocular disease. The donors’ age ranged from 9 to 93 years old. We identified genes differentially expressed with age using the R package limma (Ritchie et al., 2015) with the linear model indicated below,


$$Y_{ij}=\alpha {\mathrm{Age}}_{i}+\beta {\mathrm{Sex}}_{i}+\gamma \mathrm{Macular}/non-{\mathrm{Macular}}_{i}+\varepsilon_{ij}$$

In this regression model, *Y_ij_* is the expression level of gene *j* in sample *i*; Age*_i_* denotes the age of sample *i*; Sex*_i_* denotes the sex of sample *i* and Macular/non-Macular*_i_* denotes the anatomical location of RPE/choroid of sample *i*.

The genes were filtered to include only factors relevant to intercellular communication. As a reference, we used the genes from the LRI database of scDiffCom (that also includes those from CellChat, as explained above). Age-associated differential expression was considered significant for adjusted *p*-values (Benjamini-Hochberg) <0.1 and absolute log_2_ fold change bigger than log_2_(1.5)/70 years.

### Senescence gene expression and intercellular communication analysis

We scored each cell from the scRNA-seq dataset and each sample from the microarray dataset in relation to the level of expressed senescence signature genes. Such signatures were obtained from a recent meta-analysis that identified genes up-and down-regulated with senescence across 20 microarray datasets (Chatsirisupachai et al., 2019). This dataset provided 1,232 senescence signature genes. As recommended in the GSEA user guide to limit gene set within 500 genes (Hanzelmann et al., 2013), we selected only genes with a q-value from the meta-analysis <0.001, and obtained 135 upregulated and 271 downregulated genes (Supplementary Tables 1, 2). The senescence score was defined as follows:


$$\begin{array}{l} \mathrm{Senescence score}=\\ \mathrm{upregulated GSEA score}–\mathrm{downregulated GSEA score}\text{,} \end{array}$$

where each term corresponds to a gene set enrichment analysis (GSEA) score computed either on each cell or sample (ssGSEA) using the upregulated, respectively downregulated, senescence signatures as gene sets. ssGSEA scores were computed from the scRNA-seq Seurat object described above by using the function enrichIt from the R package escape (version 1.1.1; Borcherding et al., 2021). ssGSEA scores for microarray samples were computed using the R package gsva (version 1.38.2; Hanzelmann et al., 2013).

## Results

### Inferring intercellular communication in the RPE/choroid

**Figure 1:**
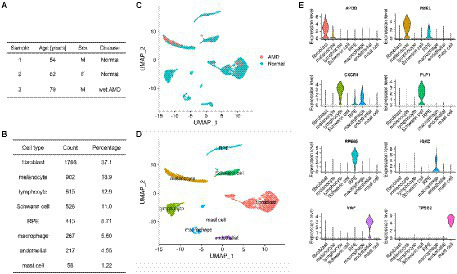
Summary of the RPE/choroid scRNA-seq dataset used for cell–cell communication analysis. **(A)** Demographic data including disease status of 3 RPE/choroid donors from GSE135922. **(B)** Count and percentage of each specific cell type detected from the 4,766 cells of the scRNA-seq data. **(C,D)** Dimensionality reduction (UMAP) of the scRNA-seq data showing eight cell types in the RPE/choroid. **(C)** Cells are colored by disease status (normal or AMD). **(D)** Cells are colored by cell-type annotations. **(E)** Violin plots showing the expression of marker genes used to annotate cell clusters.

To initiate the analysis of the intercellular communication occurring at the level of the RPE and choroid, we used RPE/choroid scRNA-seq data from the publicly available dataset GSE135922 (Voigt et al., 2019) that had been obtained from post-mortem tissues of 3 donors, including one patient with neovascular (wet) AMD and two non-AMD patients (Figure 1A). Following standard pre-processing (see Materials and methods), we annotated 4,766 cells based on previously reported cell-type-specific marker genes as follows: fibroblasts (APOD), melanocytes (PMEL), lymphocytes (CXCR4), Schwann cells (PLP1), RPE (RPE65), macrophages (IGKC), endothelial cells (VWF) and mast cells (TPSB2; Figures 1B–E). Our annotation is similar to the original work from Voigt et al. (2019), although they have described more specific clusters such as subpopulations of Schwann cells and lymphocytes that are not relevant to our current study. We next applied the CellChat algorithm to the healthy and AMD samples separately and detected 3,600 and 3,430 cell-type to cell-type interactions (CCIs), respectively (Supplementary Tables 3, 4). As it has been recently pointed out that different intercellular communication algorithms might be prone to returning different results (Dimitrov et al., 2021, 2022), we also used a second package, scDiffCom, to further confirm CellChat findings. When using the same prior LRI database as CellChat, scDiffCom returned 2,597 and 2,081 CCIs in normal and AMD samples, respectively. Out of those, 2,274 and 1827 CCIs, respectively, were commonly detected by both packages (Supplementary Figures 1A–C), indicating that the scDiffCom algorithm is generally more conservative. As expected, when using its extended curated database including more LRIs than those present in CellChat, scDiffCom returned significantly more CCIs, namely 8,629 and 7,176, respectively, (Supplementary Tables 5, 6). The detected interactions provide a global atlas of intercellular communication in the human RPE/choroid. Although we considered normal and AMD tissues separately, we note that a sample size of only three patients was not large enough to perform a complete differential ICC analysis.

### Secreted proteins-mediated signaling and extracellular matrix interactions

**Figure 2:**
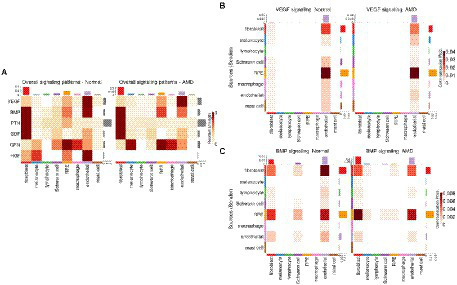
Secreted proteins-mediated signaling involving RPE cells derived from CellChat results. **(A)** Heatmap showing the relative strength of selected signaling pathways across cell types in both normal and AMD tissues in the scRNA-seq dataset from Voigt et al. (2019). Pathways were selected as those being the most relevant to communication patterns involving RPE cells. **(B)** Cell-type to cell-type heatmap of CellChat communication probabilities aggregated from each VEGF LRI. VEGF signaling was predominantly targeting endothelial cells and was especially elevated when originating from RPE cells. **(C)** Cell-type to cell-type heatmap of CellChat communication probabilities aggregated from each BMP LRI. BMP signaling was predominantly targeting fibroblasts and endothelial cells and was especially elevated when originating from RPE cells and fibroblasts.

As the RPE plays an important role in the homeostasis of surrounding tissues, we were particularly interested in secreted proteins-mediated signaling pathways engaging RPE and/or other cells. Network centrality analysis on CellChat data revealed that vascular endothelial growth factor (VEGF), bone morphogenic proteins (BMP), pleiotrophin (PTN), growth differentiation factors (GDF), granulin (GRN) and hepatocyte growth factor (HGF) were among the top such pathways between RPE cells and cells from the choroid (Figure 2A; Supplementary Figures 2A–F). The CellChat cell-type-specific networks of VEGF and BMP (Figures 2B,C) showed that RPE cells are the prominent source of secretion of effectors for these two pathways whereas endothelial cells and, respectively, fibroblast/endothelial cells are the major targets. Results derived from scDiffCom also confirmed these findings (Supplementary Figures 3A,B). VEGF denotes a group of signal proteins that promote blood vessel formation or angiogenesis (Senger et al., 1983; Ferrara et al., 2003; Gogat et al., 2004) that have been studied in various diseases (Ellis and Hicklin, 2008) as their main inhibitor (anti-VEGF) could reduce the severity of many pathological conditions, including wet AMD (Schmidt-Erfurth et al., 2014). BMP are cytokines related to tissue regeneration and development (Sieber et al., 2009). BMP has been associated with the development of the neural retina and RPE (Hocking and McFarlane, 2007), RPE migration and maintenance of RPE barrier integrity (Ibrahim et al., 2020). Here, our cell–cell communication analysis highlighted VEGF and BMP as dominant signaling pathways from the RPE to the choroid.

**Figure 3:**
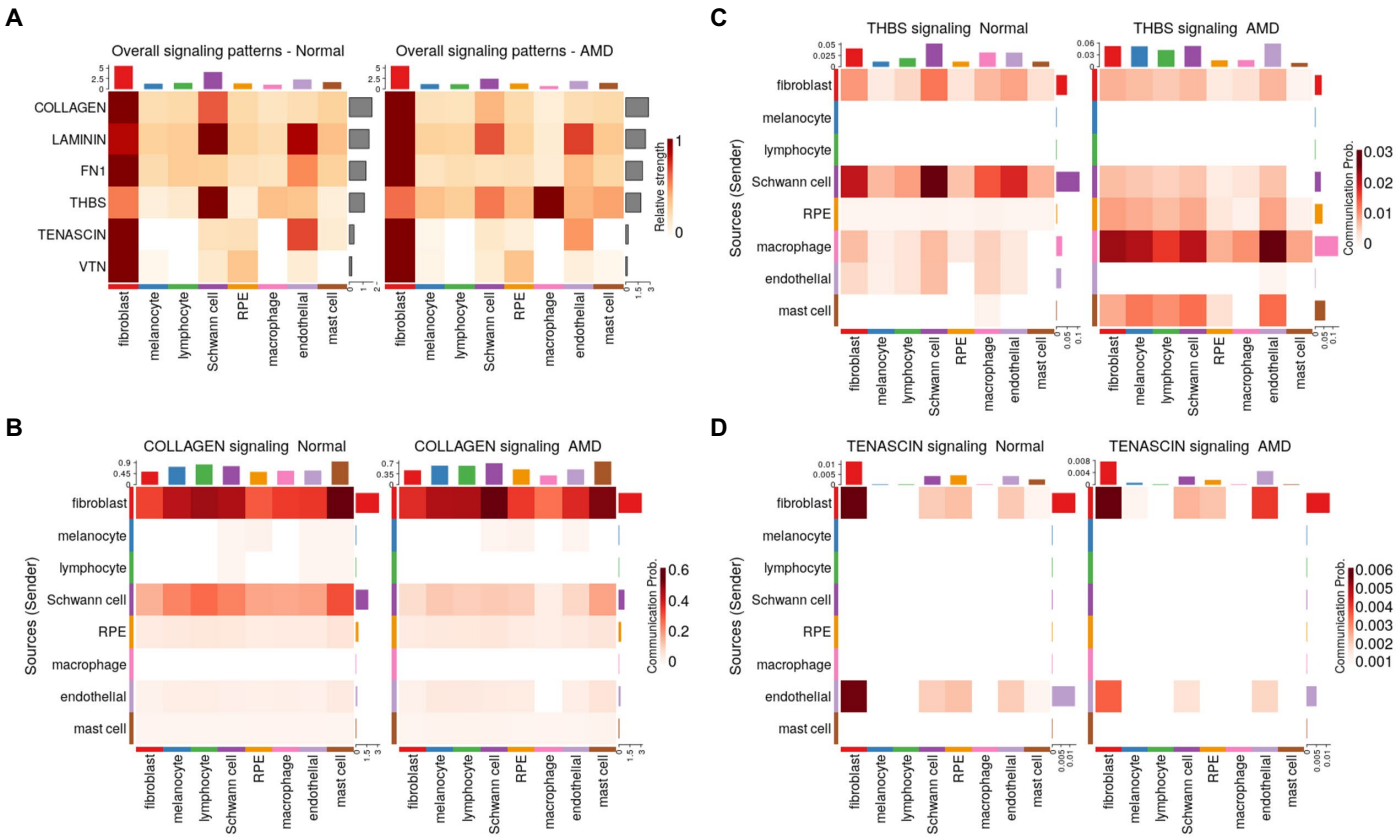
ECM-related signaling in the RPE/choroid derived from CellChat results. **(A)** Heatmap showing the relative strength of highly expressed ECM-related pathways across cell types in both normal and AMD tissues in the scRNA-seq dataset from Voigt et al. (2019). **(B)** Cell-type to cell-type heatmap of CellChat communication probabilities aggregated from each COLLAGEN LRI. COLLAGEN signaling was mostly originating from fibroblast and Schwann cells targeting all cell types similarly. **(C)** Cell-type to cell-type heatmap of CellChat communication probabilities aggregated from each thrombospondin LRI. Thrombospondin signaling was mostly originating from fibroblast and Schwann cells and targeting various cell types. **(D)** Cell-type to cell-type heatmap of CellChat communication probabilities aggregated from each tenascin LRI. Tenascin signaling was mostly originating from fibroblast and endothelial cells targeting predominantly themselves as well as RPE and Schwann cells to a lesser extent.

As alterations of the extracellular matrix have been associated with dysfunction of the RPE/choroid and with AMD, especially in relation to migration/wound healing and angiogenesis (Pouw et al., 2021), we then extracted all CCIs detected by CellChat and scDiffCom involving ECM-receptor interactions. Prominent ECM-related pathways included collagen, laminin, fibronectin 1 (FN1), thrombospondin (THBS), tenascin and vitronectin (VTN; Figure 3A). CellChat cell-type-specific networks showed that fibroblasts, Schwann cells and endothelial cells were the major sources of effectors for these pathways (Figures 3B–D). Again, the same patterns were revealed by scDiffCom (Supplementary Figures 4A–C).

### Age-associated ligands and receptors in the RPE/choroid

As there is currently no scRNA-seq dataset of the RPE/choroid across the human lifespan, we relied on the bulk microarray data from (Newman et al., 2012) to investigate potential age-related expression changes in ligand and receptor genes. We performed a linear regression across age and accounted for potential confounding factors such as retinal location (macular versus non-macular). Out of the 1,854 unique ligand/receptor genes present in the scDiffCom LRI database, we found that 60 of them were upregulated and, respectively, 103 of them were downregulated, with age in the bulk data (BH adjusted *p*-value < 0.1 and|log_2_ (fold change)| > log_2_(1.5)/70 years; Supplementary Table 7). To focus on the communication between the RPE and the cell types of the choroid, we then selected the genes that were explicitly taking part in a least one cell–cell interaction detected by scDiffCom either originating from or targeting the RPE. We obtained 46 and 43 ligand/receptor genes upregulated and, respectively, downregulated with age (Table 1).

**Table 1:** Ligands and receptors that are differentially expressed with age in RPE/choroid microarray samples and taking part in cell–cell interactions (detected by scDiffCom in scRNA-seq data) specifically from or to the RPE cell type.

Ligands				Receptors			
Regulation	Gene	log2FC	Adj. value of *p*	Regulation	Gene	log2FC	Adj. value of *p*
UP	AREG	0.020	0.061	UP	APLP1	0.011	0.005
	BMP7	0.019	0.032		ATP1A3	0.008	0.010
	BSG	0.011	0.012		EZR	0.015	0.022
	C1QTNF5	0.012	0.032		F2RL2	0.024	0.015
	CDH3	0.011	0.067		FAS	0.010	0.025
	CIRBP	0.013	0.010		FGFRL1	0.010	0.017
	CNTN3	0.015	0.044		GPC1	0.010	0.001
	COL20A1	0.017	0.007		INSR	0.009	0.021
	COL8A1	0.013	0.024		ITGA6	0.008	0.084
	EFNA2	0.009	0.005		ITGB8	0.027	0.008
	FNDC5	0.012	0.032		LRP8	0.016	0.024
	GDF11	0.012	0.069		LSR	0.012	0.017
	MYOC	0.020	0.025		MERTK	0.009	0.025
	OMG	0.016	0.095		NETO2	0.023	0.007
	PTN	0.009	0.096		PTPRZ1	0.011	0.063
	SAA1	0.021	0.048		SCARB1	0.009	0.007
	SERPINF1	0.013	0.055		SDC4	0.010	0.056
	SFRP1	0.024	0.008		SLC16A1	0.016	0.010
	SPON1	0.013	0.051		STRA6	0.014	0.051
	SPTBN2	0.015	0.002		TRPM3	0.021	0.010
	THBS2	0.012	0.090		VASN	0.017	0.002
	THBS4	0.017	0.011	DOWN	ADRB2	−0.009	0.019
	TTR	0.025	0.016		AR	−0.010	0.010
	VEGFA	0.012	0.002		BAMBI	−0.011	0.002
	ZP3	0.009	0.078		BMPR2	−0.010	0.006
DOWN	ADM	−0.011	0.048		EPHA4	−0.009	0.069
	ANGPT1	−0.027	0.000		FZD2	−0.008	0.021
	COL3A1	−0.013	0.010		FZD8	−0.009	0.063
	CXCL12	−0.012	0.013		HHIP	−0.012	0.032
	EFNB2	−0.011	0.002		IGF2R	−0.011	0.030
	FBN1	−0.011	0.021		IL6ST	−0.009	0.032
	FGF12	−0.014	0.010		ITGA9	−0.009	0.044
	FSTL1	−0.008	0.051		JAML	−0.012	0.055
	HBEGF	−0.010	0.051		KDR	−0.011	0.025
	JAG1	−0.009	0.005		KIT	−0.009	0.095
	LTB	−0.011	0.064		KLRG1	−0.011	0.003
	NRG1	−0.012	0.022		NRP1	−0.012	0.002
	RSPO3	−0.020	0.001		NRP2	−0.011	0.017
	S100A4	−0.011	0.010		PLXNA4	−0.012	0.053
	SEMA3E	−0.008	0.057		PRTG	−0.009	0.010
	SEMA5A	−0.011	0.006		PTPRB	−0.010	0.069
	SLIT2	−0.011	0.007		TLR4	−0.009	0.069
	SLIT3	−0.011	0.002				
	TF	−0.013	0.032				
	TNFSF10	−0.009	0.049				
	TNFSF13B	−0.013	0.012				
	TNXB	−0.014	0.004				

**Figure 4:**
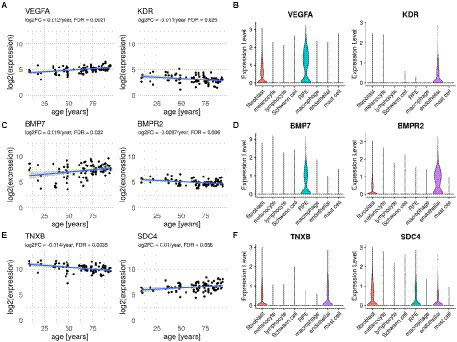
Ligand and receptor genes differentially expressed with age in the RPE/choroid with their typical cell type-specific expression. **(A,C,E)** Scatter plots of selected ligands and receptors showing expression changes with age in human RPE/choroid microarray samples from Newman et al. (2012). Displayed Log2FC and FDR are those computed with Limma according to the linear model described in Methods. **(B,D,F)** Violin plots showing expression levels of corresponding genes in RPE/choroid cell types from scRNA-seq samples of Voigt et al. (2019).

Several of the genes differentially expressed with age were part of the top secreted pathways detected by CellChat/scDiffCom from or to RPE cells, including *VEGFA*, *KDR*, *BMP7*, *BMPR2*, *PTN*, *SDC4* and *GDF11* (Table 1). Regarding VEGF pathway, the ligand gene *VEGFA* was upregulated with age in RPE/choroid bulk data (Figure 4A) and mainly expressed by RPE cells (Figure 4A) in scRNA-seq data. *KDR*, a receptor of the VEGF pathway, was downregulated with age in RPE/choroid bulk data (Figure 4A) and predominantly expressed by endothelial cells in scRNA-seq data (Figure 4B). Regarding the BMP pathway, the ligand gene *BMP7* was upregulated in aging RPE/choroid bulk data (Figure 4C) and mainly expressed by RPE cells (Figure 4D) in scRNA-seq data. *BMPR2*, a receptor of the BMP pathway, was downregulated with age in RPE/choroid bulk data (Figure 4C) and mainly expressed by endothelial cells (Figure 4D). Collectively, these results support the idea that aging RPE cells tend to increase the secretion of VEGF and BMP factors toward the choroid, whereas endothelial cells tend to decrease the expression of the corresponding receptors, suggesting a potential compensatory mechanism, which may offer some protection against initial pathological changes.

Several genes belonging to the top ECM-related pathways detected by CellChat from the scRNA-seq data were also differentially expressed with age in the bulk dataset, including collagen and integrin genes, *THBS2*, *THBS4*, *SDC4* and *TNXB* (Table 1). Among these, the ligand-receptor pair TNXB/SDC4 was particularly noteworthy. *TNXB* was expressed mostly by fibroblasts and endothelial cells in the scRNA-seq data (Figure 4F) and downregulated with age in the bulk data (Figure 4E). *SDC4* was expressed by RPE cells, fibroblasts and endothelial cells in the scRNA-seq data (Figure 4F) and upregulated with age in the bulk data (Figure 4E). Changes in tenascin expression have been previously related to development and aging (Choi et al., 2020; Matsumoto and Aoki, 2020) as well as to pathologic conditions such as cancer, inflammation and fibrosis (Albacete-Albacete et al., 2021; Tajiri et al., 2021). Disruption of the tenascin pathway also results in promoting inflammatory processes and angiogenesis (Kobayashi et al., 2016). Tenascin pathway genes were also reported as being enriched among differentially expressed genes in the RPE/choroid in AMD, including Tenascin-C (Dhirachaikulpanich et al., 2020) and *TNXB* (Porter et al., 2019).

### Effects of cellular senescence on the RPE/choroid intercellular communication

**Figure 5:**
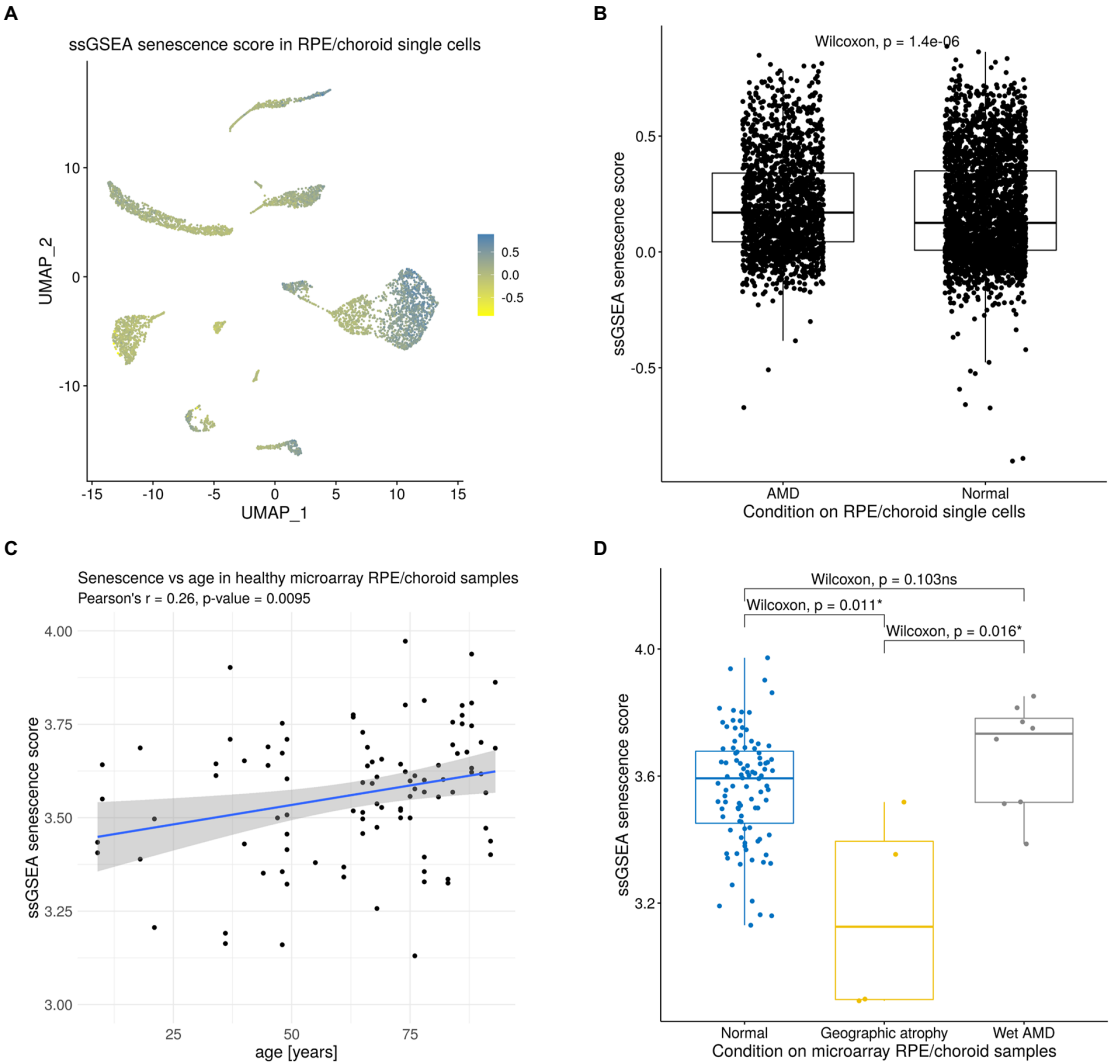
Expression of senescence signature genes in RPE/choroid scRNA-seq and microarray data. **(A)** Dimensionality reduction (UMAP) showing the ssGSEA senescence score of each cell in the scRNA-seq dataset from Voigt et al. (2019). **(B)** Boxplot showing a slight increase in the senescence score of AMD cells compared to normal cells from Voigt et al. (2019). **(C)** Positive correlation between senescence scores and age in the non-AMD microarray samples of Newman et al. (2012) (Pearson’s correlation *R* = 0.26, value of *p* = 0.0095). **(D)** Boxplot showing significant and non-significant differences in senescence scores between normal, wet AMD and geographic atrophy RPE/choroid samples from Newman et al. (2012).

We next sought to characterize potential cellular senescence signatures in the RPE/choroid as well as their effects on intercellular communication. We first assigned a senescence score to each cell of the RPE/choroid scRNA-seq dataset from Voigt et al. (2019), using the ssGSEA approach (Barbie et al., 2009; Borcherding et al., 2021). As reference signatures, we used the results from a previous meta-analysis that inferred genes up-and down-regulated with senescence by comparing 20 bulk transcriptomics datasets (Chatsirisupachai et al., 2019; Supplementary Tables 1, 2). The senescence score was defined as the difference between the ssGSEA score of upregulated signature genes and the ssGSEA score of downregulated signature genes (Figure 5A). We noticed that cells from the patient with AMD had a slightly larger average senescence score (mean = 0.21, SD = 0.21) compared to cells from healthy samples (mean = 0.18, SD = 0.22; Wilcoxon Rank Sum Test, value of *p* = 1.37E-6; Figure 5B). However, more data with a larger sample size in terms of patients will be required in the future to better establish if AMD is consistently associated with an increased expression of senescence-related genes.

We then used the senescence score to label cells as either “normal” or “senescent-like.” It is important to note that this method did not allow us to claim with certainty that cells with high scores were actually in a senescent state before being captured for sequencing. Nevertheless, it allowed us to extract cells that at least showed a senescence-like gene expression profile. In practice, we defined such cells as those having a score in the top 20% of all senescence scores across all cell types (Supplementary Figures 5A–C). We did not find any relevant differences in the distribution of the number of such senescent-like cells across cell types between the three donors. However, we confirmed that the expression of genes involved in typical senescence pathways, including *TP53*, *CDKN1A*, *RB1*, *NFKB1* and *NOTCH1*, was generally higher in senescent-like cells compared to normal cells (Supplementary Figure 5D).

**Figure 6:**
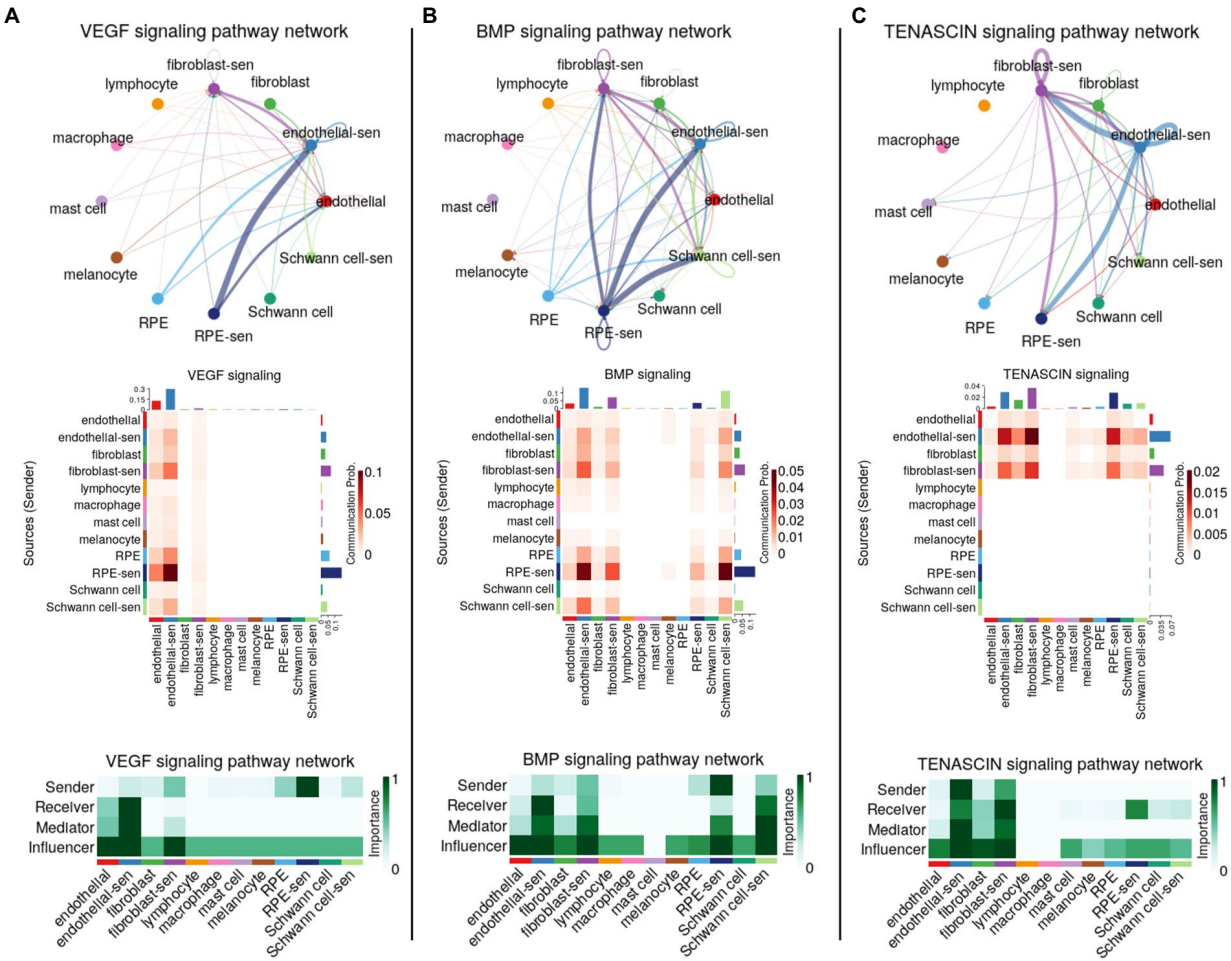
Network representation of the impact of senescence on VEGF, BMP and TENASCIN intercellular communication in the RPE/choroid. Signaling network summary between senescent-like and non-senescent cell types for **(A)** VEGF, **(B)** BMP and **(C)** TENASCIN pathways. Cell type to cell type networks (top panels) and heatmaps (middle panels) show the CellChat communication probability (aggregated over all LRIs from the given pathway) between each cell type pair. Centrality heatmaps (bottom panels) show the various CellChat centrality scores across each cell type for the selected pathways.

We performed a new intercellular communication analysis on the same scRNA-seq data as above by splitting each cell type into the two subpopulations of normal and senescent-like cells (and by merging the samples of all three donors together). As we did not consider groups with <11 cells, the analysis was performed on 12 subpopulations (Supplementary Figure 5C). When using the LRI database from Cellchat, CellChat and scDiffCom returned, respectively, 11,162 and 5,637 CCIs, out of which 5,044 were detected by both methods (Supplementary Figures 6A; Supplementary Tables 8, 9). When using its extended LRI database, scDiffCom returned 18,908 CCIs (Supplementary Table 9). Ranking pathways according to centrality measures with CellChat revealed that the pathways previously found to be associated with aging were among the top signaling patterns associated with senescent cell subpopulations. VEGF was predominantly expressed from senescent-like (sl-) RPE cells toward sl-endothelial cells (Figure 6A). Similarly, BMP showed stronger signaling from sl-RPE cells toward sl-endothelial cells, sl-Schwann cells and sl-fibroblasts compared to all other cell types (Figure 6B). Finally, tenascin-mediated signaling was also stronger between sl-fibroblasts and sl-endothelial cells and from these two cell populations toward sl-RPE cells (Figure 6C).

Results from scDiffCom generally recapitulated most of these findings from CellChat, with some differences to mention. First, scDiffCom did not predict as CellChat that sl-endothelial cells were more targeted by VEGF than endothelial cells. Instead, these two cell populations shared similar overall “receiver scores” (Supplementary Figure 6B). We still noted, in agreement with CellChat, that VEGF was secreted more by sl-RPE cells than by RPE or other cells. Second, scDiffCom did not predict a strong BMP signaling from sl-RPE cells toward sl-Schwann cells, but instead toward Schwann cells (Supplementary Figure 6C). Finally, although scDiffCom predicted as CellChat a strong tenascin signaling from sl-endothelial cells toward sl-fibroblasts, the opposite signaling from sl-fibroblasts toward sl-endothelial cells was much weaker than as predicted by CellChat (Supplementary Figure 6D). Also more generally, the overall decrease of tenascin signaling involving fibroblasts compared with sl-fibroblasts appeared less prominent when looking at scDiffCom compared to CellChat analysis.

Despite these differences, the results from both CellChat and scDiffCom analyses highlighted how senescence state might influence RPE/choroid tissue in both normal and AMD, specifically by enhancing ICC *via* VEGF, BMP and tenascin pathways.

### Effects of age on cellular senescence scores in microarray RPE/choroid data

As we showed that senescence might influence ICC in the RPE/choroid, we further explored other potential factors that might be related to senescence in this tissue. To test the hypothesis that senescence might increase with age in the RPE/choroid, we assigned a senescence score to each sample of the bulk microarray dataset from Newman et al. (2012) and explored its evolution with time. We found that the senescence score was significantly correlated with age (Pearson’s correlation *R* = 0.26, value of *p* = 0.0095; Figure 5C). This is consistent with earlier observations reporting a similar accumulation of senescent cells in different other tissues (Chatsirisupachai et al., 2019). We also compared the senescence score between control (non-AMD) samples and different types of AMD, including neovascular AMD and geographic atrophy (an end-stage non-neovascular type AMD). The results showed that neovascular AMD samples (*n* = 8) had a slightly, but non-significantly, higher senescence score than non-AMD ones (*n* = 96; Wilcoxon Rank Sum Test, value of *p* = 0.103), while geographic atrophy samples (*n* = 4) had a significantly lower senescence score than non-AMD samples (*n* = 96; value of *p* = 0.011) and neovascular AMD samples (*n* = 8; value of *p* = 0.016; Figure 5D). Overall, these two analyses suggested that the senescence score in the RPE/choroid may be affected by both age and AMD types. However, the fact that when comparing non-AMD to wet AMD there is no significant difference in the bulk microarray dataset (Figure 5D) but a significant difference in the single-cell dataset (Figure 5B) shows that further studies leading to more data and further analyses are necessary. Nevertheless, the fact that the RPE/choroid cells with high senescence scores presented increased signaling *via* VEGF, BMP and tenascin pathways suggests a possible connection between senescence and AMD RPE/choroid.

## Discussion

An increasing number of patients suffer from the main age-related degenerative eye disease involving retinal tissues, AMD, which may lead to irreversible blindness. Current standard treatments can only address a small subset of the disease by slowing down the disease progression (Wong et al., 2014; Mitchell et al., 2018), highlighting the importance and need for better understanding the biology of the RPE/choroid tissue in the context of aging and cellular senescence (Sreekumar et al., 2020; Lee et al., 2021). At the histological level, it is known that aging leads to abnormality of pigmentation with increase of soft drusen (a lipoproteinaceous deposit) between the RPE and the underlying choroid (Boulton et al., 2004; Curcio et al., 2009; Ardeljan and Chan, 2013; Curcio, 2018). At molecular level, a recent first RNA-seq global gene expression study of the aging human RPE reported upregulation of the visual cycle genes in the RPE with increasing age (Butler et al., 2021) and bulk and single-cell gene expression analyses of the aging human choriocapillaris reported an increase of the pro-inflammatory environment in the choroid (Voigt et al., 2020).

The study presented herein focused on the communication occurring at the level and between these two tissues, and on the effect that the aging process has on this communication. By applying algorithms that have proved useful to study intercellular communication in various tissues from scRNA-seq data (Armingol et al., 2021; He et al., 2021; Hu et al., 2021; Jin et al., 2021; Lagger et al., 2021), we were able to extract important signaling patterns between the RPE and the choroid. Together with our aging and cellular senescence analyses, our analysis predicted three pathways (VEGF, BMP and tenascin) that support some of the strongest RPE/choroid cell–cell interactions, while being significantly affected by age and enhanced between subpopulations of cells showing a senescent-like gene expression profile.

The analysis of secreted molecules with roles in cell signaling revealed age-related changes in communication involving VEGF and BMP. VEGF signaling has been studied extensively in many retinal diseases, especially AMD and diabetic retinopathy (Marneros, 2016). Treatments targeting VEGF can slow down the progression of neovascular AMD, despite not reversing it (Schmidt-Erfurth et al., 2014). VEGFA is secreted basolaterally from the RPE and interacts with VEGF receptors such as KDR and VEGFR-2 (Blaauwgeers et al., 1999; Gogat et al., 2004; Hocking and McFarlane, 2007). By combining our aging analysis on bulk microarray samples with cell-type-specific gene expression knowledge extracted from scRNA-seq data, we suggested that aging correlated with increased *VEGFA* expression in RPE cells and with decreased expression of the *KDR* in choroidal endothelial cells. Similarly, we identified BMP as another signaling pathway of the RPE/choroid affected by aging. BMP signaling is involved in cell regulation processes, including cell proliferation, differentiation and eye morphogenesis (Solursh et al., 1996; Ibrahim et al., 2020). BMP7 plays a role in epithelial-mesenchymal transition, angiogenesis, and antifibrotic activity (Yang et al., 2020). A previous study showed that BMP7 could reduce proliferative vitreoretinopathy, a major complication of end-stage retinal detachment, by inhibiting RPE cells’ fibrosis in a rabbit model (Yao et al., 2019). Our findings indicated an increase in *BMP7* expression in RPE cells and a decreased expression of the *BMPR2* receptor in choroidal endothelial cells during human aging. This result underscores the importance of the BMP signaling pathway for the homeostasis of the RPE/choroid. Although it is unclear why the expression of the receptor and ligand genes change in opposite directions, we hypothesized that it could be a protective adaptation to maintain the overall activity level of these two signaling pathways during aging. Although available data do not provide information about the precise dynamics of molecular interactions between ligands and receptors, we suggest that a decrease in receptor expression might be a compensatory response to maintain a relatively stable level of signaling when facing an increase in ligand concentration. It has also been reported that choriocapillaris coverage of Bruch’s membrane decreases with age (Ramrattan et al., 1994; Lutty et al., 2020), while some reports point toward changes in the number of RPE cells during aging (Gao and Hollyfield, 1992; Harman et al., 1997; Del Priore et al., 2002; Ach et al., 2014). Whether compensatory gene expression occurs in aging in order to maintain an appropriate level of cellular signaling needs to be investigated further experimentally.

Age-associated changes in the extracellular matrix of the RPE/choroid have been observed in both fundus and post-mortem tissues (Ardeljan and Chan, 2013; Boulton, 2013; Sura et al., 2020). Typically, AMD is associated with a thickening of the RPE basal lamina-Bruch’s membrane complex driven by the accumulation of basal laminar deposit (Sura et al., 2020). Genes associated with ECM were also reported to be altered in AMD, including factors regulating wound healing and angiogenesis (Pouw et al., 2021). Here, our analysis suggested that the tenascin-mediated pathway might have an age-related significance to the RPE/choroid intercellular communication. The tenascin pathway contributes to ECM maintenance (Reinhard et al., 2017; Matsumoto and Aoki, 2020; Miller, 2020) and the absence of *TNXB* was reported to cause a reduction of collagen and of tissue strength (Mao et al., 2002). Tenascin has also a proangiogenic effect when interacting with the VEGF pathway (Ikuta et al., 2000). Our group previously identified *TNXB* as a methylation target that shows a decrease in methylation in its exon3 in RPE/choroid affected by AMD (Porter et al., 2019). Genome-wide association studies (GWAS) previously identified *TNXB* as a genetic variant associated with AMD (Cipriani et al., 2012; Fritsche et al., 2016). Our current analysis suggested that *TNXB* is expressed by choroidal fibroblasts and endothelial cells and is globally downregulated with age while its receptor *SDC4* is expressed by fibroblasts, RPE cells and endothelial cells and is globally upregulated with age. This again suggested a potential compensatory mechanism. Together, our transcriptomic analysis and previous studies on DNA methylation and genetic variants emphasized the importance of *TNXB* and the tenascin pathway on the pathological mechanisms associated with aging and AMD, especially in relation to ECM interactions between the RPE and the choroid affected by AMD.

We acknowledge that this current analysis has several limitations. Firstly, the single-cell data we retrieved contains only provided 3 RPE/choroid samples: two from normal eyes and one from an eye with neovascular AMD (Voigt et al., 2019). To the best of our knowledge, there are currently no other public datasets thatcontain more samples suitable for our analysis. For example, although a recent scRNA-seq dataset from Voigt et al. (2022) contains more eye samples, it is not suitable for our intercellular communication study. Specifically, as cells have been sorted based on CD31 expression to enable the capture of endothelial cells, only a low number of RPE cells are present in the dataset and they are moreover confounded with photoreceptors (Voigt et al., 2019, 2022). Secondly, the bulk microarray dataset that we used for our aging-related analysis is missing clinical details regarding the post-mortem normal eyes (Newman et al., 2012). The only quality control that is provided is RNA integrity but not the detailed pathology of these eyes. Along the same line, both the scRNA-seq data (Voigt et al., 2019) and the bulk microarray data (Newman et al., 2012) are missing a clear nomenclature of neovascular AMD. The diagnosis of neovascular AMD was acquired from ophthalmic notes for the scRNA-seq dataset, while the diagnosis for the bulk microarray dataset was obtained from retina specialists and fundus photographs. However, the current clinical nomenclature (Jung et al., 2014; Spaide et al., 2020) indicates that specific subtypes of neovascularization, such as intraretinal neovascularization, might have different gene expression profiles. This emphasizes the importance of improving the clinical documentation of recovered samples in future experiments and data collections. Thirdly, a common limitation of both the single-cell and bulk datasets we analyzed is the absence of detailed spatial information. As samples originate from 8-mm tissue punches, details regarding regional differences such as between the fovea, parafovea and perifovea are lost. Indeed, several studies such as some based on imaging techniques have illustrated the importance of the topographic nature of AMD. For example, a higher concentration of melanolipofuscin is found in foveal RPE (Bermond et al., 2020). Other studies using optical coherence tomography suggest that hyperreflective foci (potentially associated with AMD progression) may be due to migrating RPE cells undergoing transdifferentiation (Ouyang et al., 2013; Cao et al., 2021), highlighting the three-dimensional nature of the disease.

Some recent studies indicate that senescent RPE cells accumulate during AMD in human donors, primates and RPE cell cultures (Wang et al., 2019; Blasiak, 2020; Lee et al., 2021), thus raising the interest in the role of cellular senescence in AMD. In addition to RPE, cellular senescence also may affect choroidal tissues and the neuronal retina (Ma et al., 2013; Cabrera et al., 2016). However, it is still unclear what are the causal role and function of the potential senescent cells in human pathology as most studies are limited to animal models and cell cultures (Cao et al., 2013; Chaum et al., 2015; Cabrera et al., 2016). Our results further highlight the importance of cellular senescence in the RPE/choroid tissues in relation to prominent cell–cell communication pathways mediated by VEGF, BMP and tenascin. These findings support potential alternative treatment approaches for AMD based on targeting senescent cells using senotherapies and senolytic drugs. The strategy to target senescent cells to prevent or slow down the progress of AMD has also been independently suggested by a recent review (Lee et al., 2021). A proposed approach is to inhibit pro-inflammatory signaling molecules and to regulate oxidative stress-induced senescence (Sreekumar et al., 2020). Notably, as our results suggested that different subtypes of AMD (geographic atrophy and neovascular AMD) may present differences in senescence status, such subtypes should be taken into consideration before applying senotherapies. To achieve this promising goal, further studies on the biological mechanisms of senescence in AMD and further clinical studies using senotherapies to treat AMD are needed (El-Nimri et al., 2020).

## Data availability statement

The datasets presented in this study can be found in online repositories. The names of the repository/repositories and accession number(s) can be found at: https://github.com/CyrilLagger/amd_aging.

## Author contributions

DD and KC conceptualized the study. DD, CL, and KC performed the analysis and wrote the first draft of the manuscript. LP conceived the overall project. LP and JM supervised the study and provided critical insights for the interpretation of results. All authors contributed to the article and approved the submitted version.

## Conflict of interest

JM is an advisor/consultant for the Longevity Vision Fund, NOVOS, Insilico Medicine, YouthBio Therapeutics and the founder of Magellan Science Ltd, a company providing consulting services in longevity science.

The remaining authors declare that the research was conducted in the absence of any commercial or financial relationships that could be construed as a potential conflict of interest.

## Publisher’s note

All claims expressed in this article are solely those of the authors and do not necessarily represent those of their affiliated organizations, or those of the publisher, the editors and the reviewers. Any product that may be evaluated in this article, or claim that may be made by its manufacturer, is not guaranteed or endorsed by the publisher.
