# Supplementary figures and images for: Intercellular communication analysis of the human retinal pigment epithelial and choroidal cells predicts pathways associated with aging, cellular senescence and age-related macular degeneration
Source: Front Aging Neurosci. 2022 Nov 3;14:1016293. doi: 10.3389/fnagi.2022.1016293 (PMC9669800; doi:10.3389/fnagi.2022.1016293)

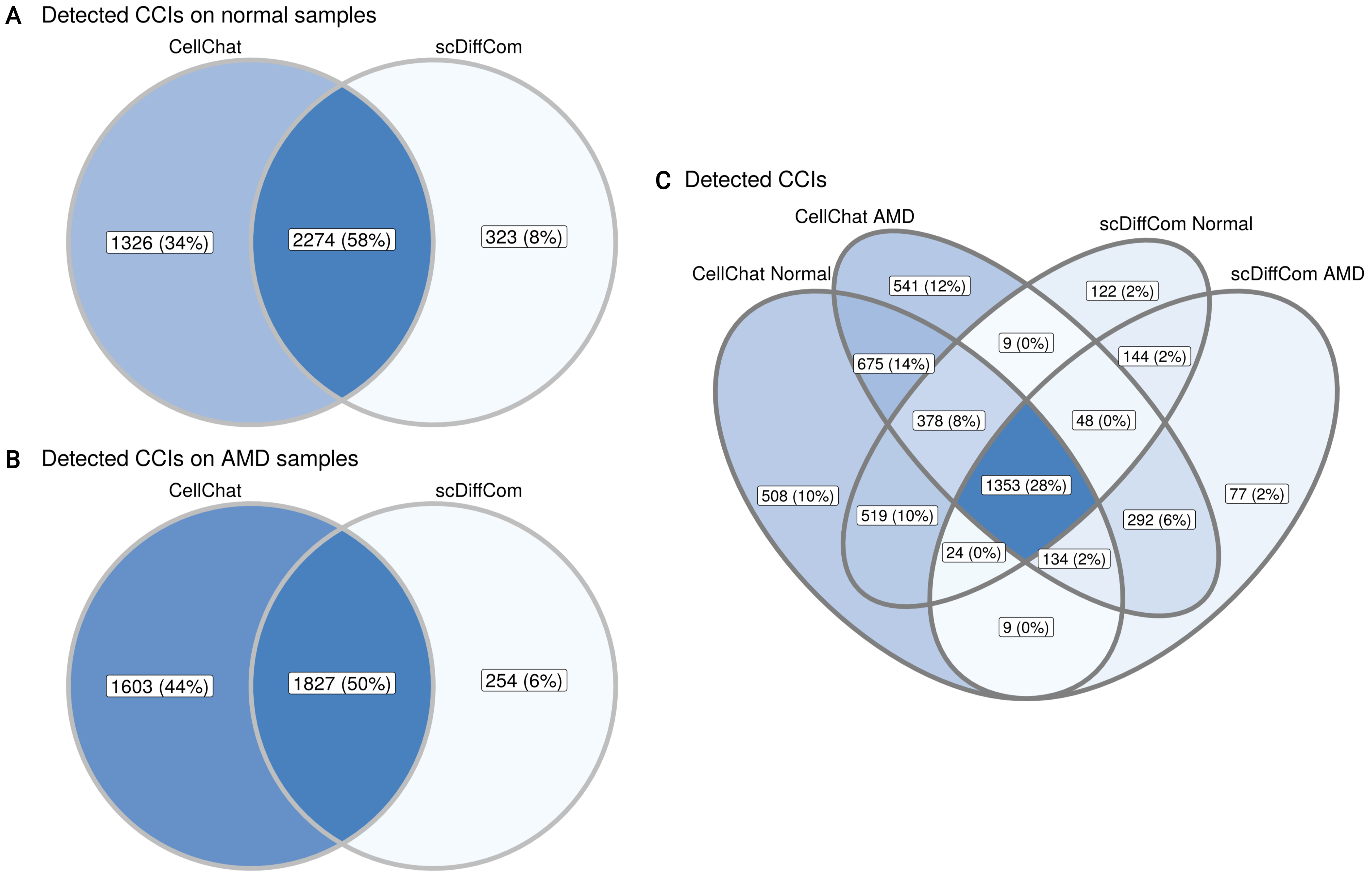

Supplement: Supplementary file 2 [file Image_1.TIFF]

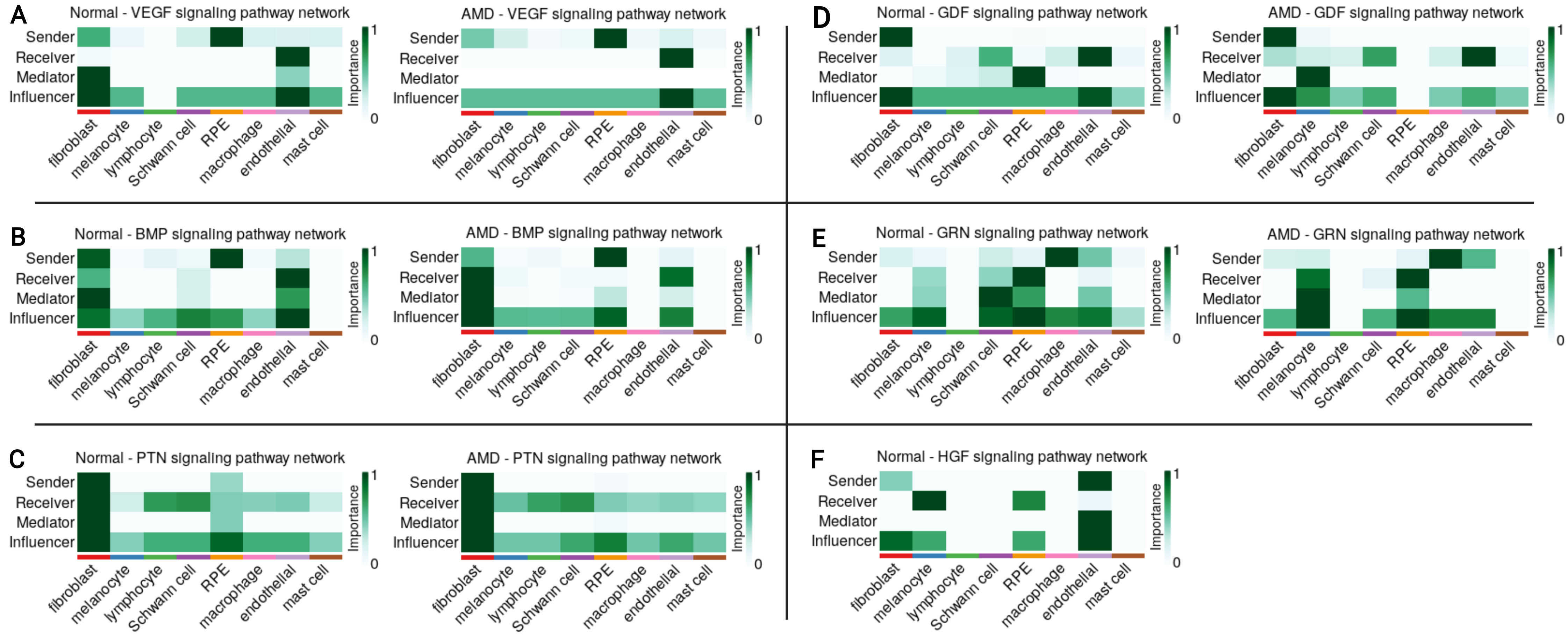

Supplement: Supplementary file 3 [file Image_2.TIFF]

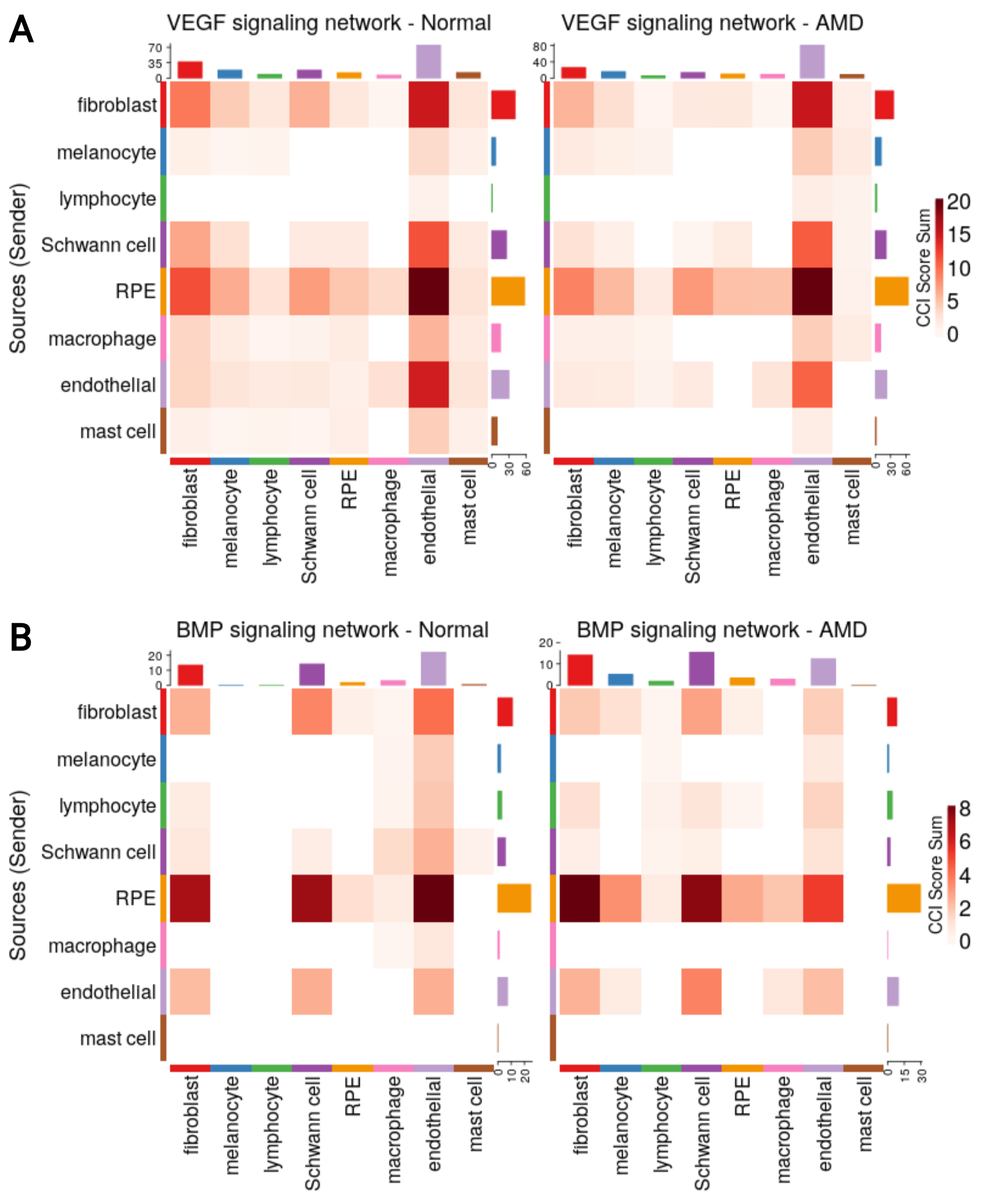

Supplement: Supplementary file 4 [file Image_3.TIFF]

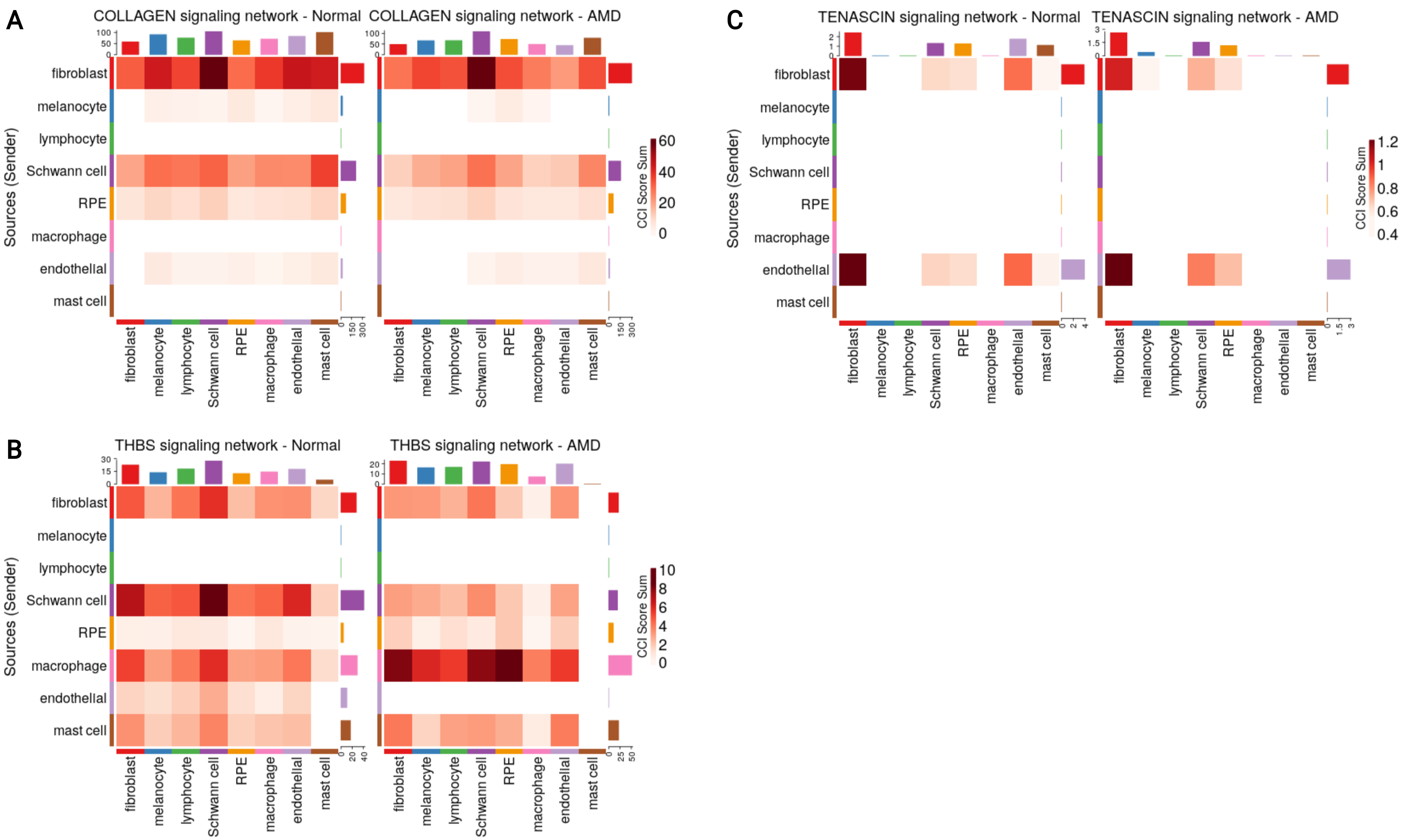

Supplement: Supplementary file 5 [file Image_4.TIFF]

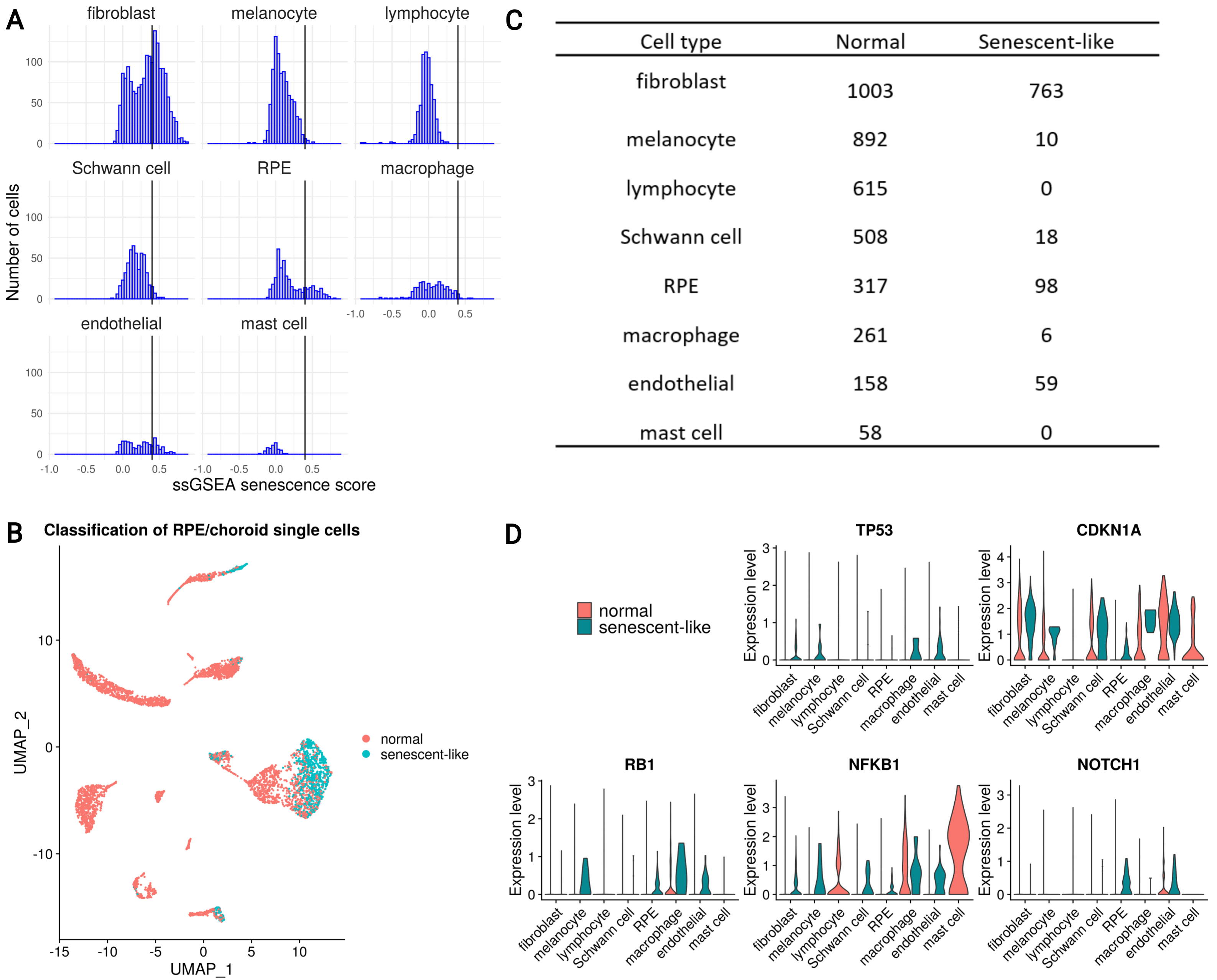

Supplement: Supplementary file 6 [file Image_5.TIFF]

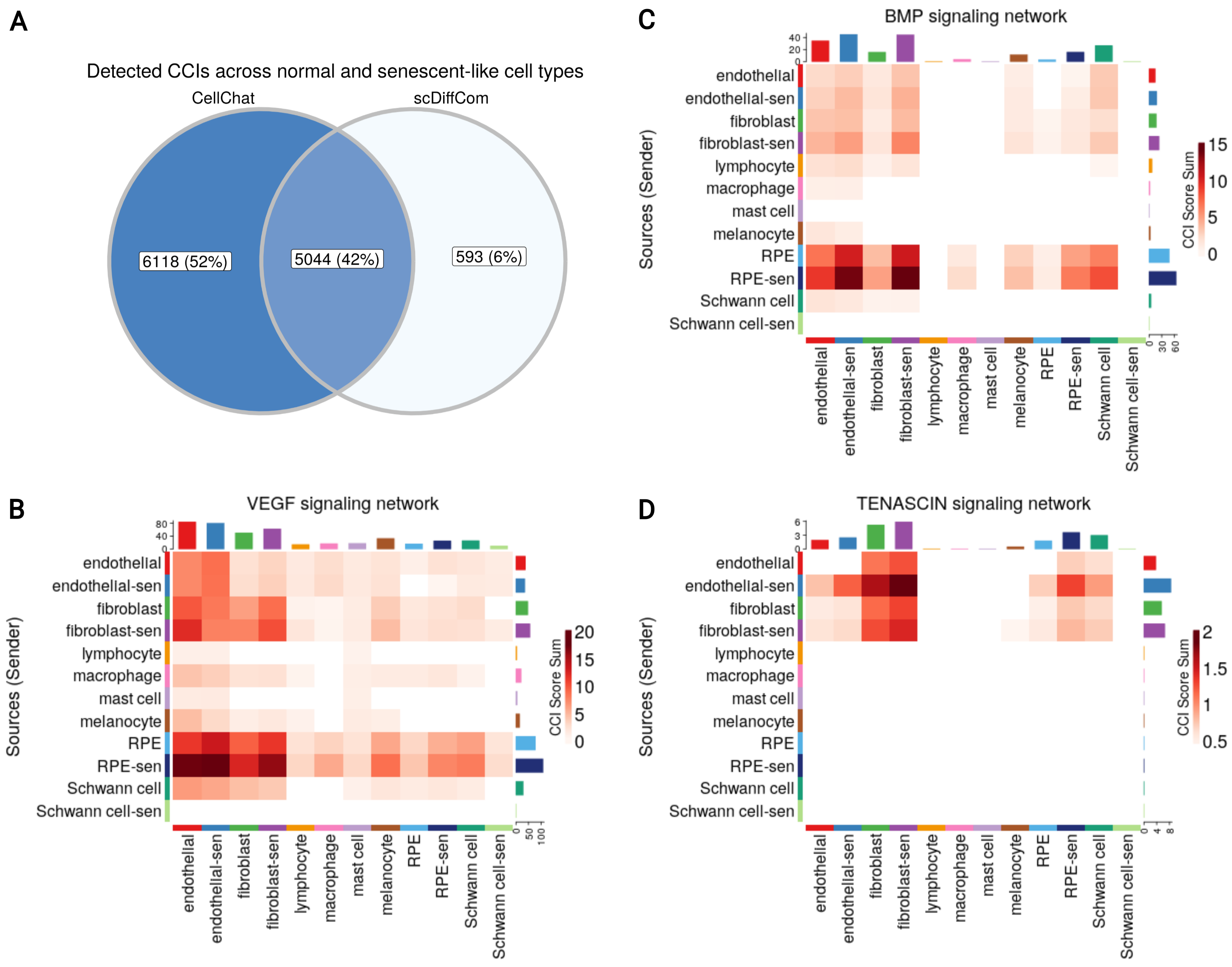

Supplement: Supplementary file 7 [file Image_6.TIFF]
